# Giving Legs to Restless Legs: A Case Study of How the Media Helps Make People Sick

**DOI:** 10.1371/journal.pmed.0030170

**Published:** 2006-04-11

**Authors:** Steven Woloshin, Lisa M Schwartz

## Abstract

To get a sense of how the media works in the context of a major disease promotion effort, the authors examined news coverage of restless leg syndrome.


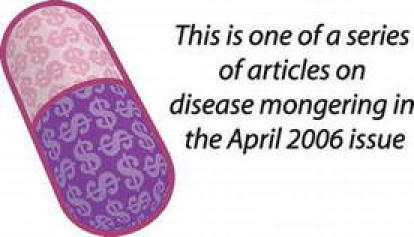

*“[Restless legs syndrome] is quite a serious sleep disorder that affects a lot of people….Their sleep is disturbed and, unless they are really awake, they will not be aware of it” [ 1].
*

Life can be hard. Sometimes you feel sad or distracted or anxious. Or maybe you feel a compelling urge to move your legs. But does that mean you are sick? Does it mean you need medication?

Maybe, maybe not. For some people, symptoms are severe enough to be disabling. But for many others with milder problems, these “symptoms” are just the transient experiences of everyday life. Helping sick people get treatment is a good thing. Convincing healthy people that they are sick is not. Sick people stand to benefit from treatment, but healthy people may only get hurt: they get labeled “sick,” may become anxious about their condition, and, if they are treated, may experience side effects that overwhelm any potential benefit.

“Disease mongering” is the effort by pharmaceutical companies (or others with similar financial interests) to enlarge the market for a treatment by convincing people that they are sick and need medical intervention [ 2]. Typically, the disease is vague, with nonspecific symptoms spanning a broad spectrum of severity—from everyday experiences many people would not even call “symptoms,” to profound suffering. The market for treatment gets enlarged in two ways: by narrowing the definition of health so normal experiences get labeled as pathologic, and by expanding the definition of disease to include earlier, milder, and presymptomatic forms (e.g., regarding a risk factor such as high cholesterol as a disease in itself).

Discussions about disease mongering usually focus on the role of pharmaceutical companies—how they promote disease and their products through “disease awareness” campaigns and direct-to-consumer drug advertising, and by funding disease advocacy groups. But diseases also get promoted in another way: through the news media. News reports are a major source of health information for people [ 3]. Unless journalists approach stories about new diseases skeptically and look out for disease mongering by the pharmaceutical industry, pharmaceutical consultants, and advocacy groups, journalists, too, may end up selling sickness.

## The Case of Restless Legs Syndrome

To get a sense of how the media works in the context of a major disease promotion effort, we examined news coverage of “restless legs” (see sidebar). In 2003, GlaxoSmithKline launched a campaign to promote awareness about restless legs syndrome, beginning with press releases about presentations at the American Academy of Neurology meeting describing the early trial results of using ropinirole (a drug previously approved for Parkinson disease) for the treatment of restless legs [ 6, 7]. Two months later, GlaxoSmithKline issued a new press release entitled “New survey reveals common yet under recognized disorder—restless legs syndrome—is keeping Americans awake at night” about an internally funded and, at the time, unpublished study [ 8]. In 2005, the US Food and Drug Administration (FDA) approved ropinirole for the treatment of restless legs syndrome (the first drug approved specifically for this indication). Since then, the restless legs campaign has developed into a multimillion dollar international effort to “push restless legs syndrome into the consciousness of doctors and consumers alike” [ 9].

## Newspaper Coverage of the Restless Legs Syndrome

To identify media coverage related to this campaign over two years (November 2003–November 2005), we did full-text searches of “major newspapers” in Lexis-Nexis and ProQuest databases and found 187 unique articles with the phrase “restless legs.” We excluded articles not about the syndrome (e.g., “Elvis's restless legs”), nonnews stories (e.g., health advice columns, notices of restless legs health screenings/support groups), and articles with only passing mention of restless legs (most of these were about sleep disorders, another “new yet largely unrecognized problem”). We analyzed the remaining 33 articles (all focused on restless legs syndrome) using an explicit coding scheme organized around the key elements of disease mongering, as outlined in the first column of Table 1: exaggerating the prevalence of the disease (e.g., uncritically accepting a broad prevalence estimate), encouraging more diagnosis (e.g., doctors fail to recognize it), and suggesting that all disease should be treated (e.g., overstating the benefits or minimizing the harms of treatment).

**Table 1:**
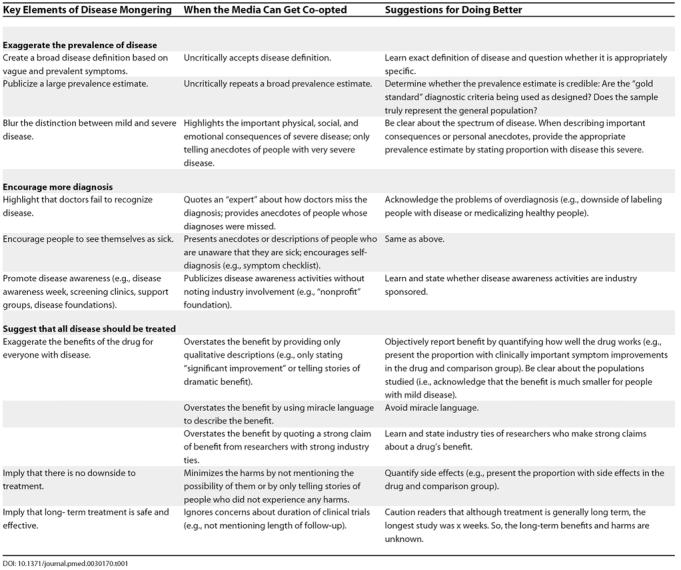
Key Elements of Disease Mongering and How the Media Could Do Better

## Exaggerating Disease Prevalence

Figure 1 shows that the news articles often included elements exaggerating disease prevalence. Only one article questioned the disease definition at all (and portrayed the act of questioning the definition as insensitive: “[the patient] knows it can sound trivial. That's one of the problems with restless legs. Radio show host Rush Limbaugh, for example, has mocked it as a pseudoillness” [ 10]).

**Figure 1:**
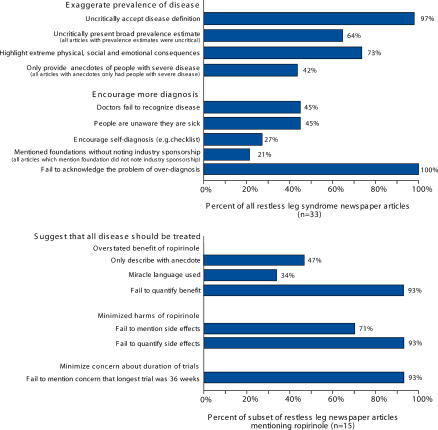
Frequency of Key Elements of Disease Mongering in Newspaper Articles Top bar graph analyzes all articles about restless legs syndrome. Bottom bar graph analyzes the subset that mentions ropinirole

Almost two-thirds of articles provided an estimate of disease prevalence (most commonly, statements such as “at least 12 million Americans suffer from the syndrome” [ 11] or “[it] affects 1 in 10 adults in the United States” [ 12]). No article questioned the validity of the prevalence estimates. In fact, there are reasons to believe the estimates overstate the prevalence of clinically meaningful disease. For example, the frequently cited 10% estimate came from a study that used a single question to identify restless legs syndrome rather than the four standard criteria [ 13]. The less stringent definition inflates the estimate because people with other causes of leg symptoms (e.g., leg cramps or diabetic neuropathy) are counted incorrectly as having the syndrome.

In a recent large study, only 7% of respondents reported all four diagnostic criteria, and only 2.7% reported moderately or severely distressing symptoms two or more times per week (i.e., the group for whom medical treatment might be appropriate) [ 14]. Even the 2.7% estimate is probably too high, because of bias inherent in the study sample. The authors claimed an implausible 98% response rate to their random-digit dial survey (typical response rates are 50%–70% [ 15]). Most likely, the authors meant that 98% of individuals who agreed to participate completed the survey. But respondents agreeing to participate in a restless legs study are more likely to have leg-related symptoms than nonrespondents.

Nearly three-quarters of newspaper articles highlighted the potentially serious physical, social, and emotional consequences of restless legs: “…the condition sounds like a joke, but its consequences can be devastating. Driven to despair by years of sleepless nights, patients have become suicidal” [ 16]). While over 40% of the articles provided anecdotes about people with severe disease, no article provided anecdotes about people who did not find their symptoms especially bothersome.

## Encourage More Diagnosis

The articles also reinforced the need for more diagnosis. About half reported that the syndrome is underdiagnosed by physicians (“…relatively few doctors know about restless legs. This is the most common disorder your doctor has never heard of” [ 17]) and underrecognized by patients (“…many people can suffer in silence for years before it is recognized” [ 18]). One-quarter of articles encouraged patient self-diagnosis and suggested people ask their doctor whether restless legs might explain various problems (including insomnia, daytime fatigue, attention deficit disorder in children, and depression). One-fifth of articles referred readers to the “nonprofit” Restless Legs Foundation for further information; none reported that the foundation is heavily subsidized by GlaxoSmithKline. No article acknowledged the possibility of overdiagnosis (the idea that some people will be diagnosed unnecessarily and take medication they do not really need).

## Suggest That All Disease Should Be Treated

About half the news stories mentioned the drug ropinirole by name. Only one story quantified the drug's benefit. By contrast, about half the stories mentioning ropinirole included anecdotes about patients who took the drug (and in most cases noted substantial improvement). One-third of articles used “miracle language” to describe patient response to medication (e.g., “it has been a miracle drug for me” [ 19]). The actual benefit of the drug is modest. The drug label reports that in a 12-week US clinical trial, restless legs symptom scores (measured on a 40-point scale) improved by 13.5 points for patients taking ropinirole compared with 9.8 points for those taking placebo [ 20]. In more clinical terms, 73% taking ropinirole responded to the drug (i.e., restless legs scores improved by six points) compared with 57% taking placebo.

The drug label [ 20] also notes that ropinirole has a number of side effects, including nausea (40% in ropinirole group versus 8% in placebo group) and dizziness (11% versus 5%, respectively). Somnolence and fatigue (ostensibly, the real target of the drug) were also higher in the ropinirole versus the placebo group (12% versus 6%; 8% versus 4%, respectively). Nonetheless, only five of the 15 articles mentioning ropinirole noted that it could have side effects and just one quantified the chance of any side effect (“nausea was the most common side effect, reported in 38% of patients” [ 18]). Finally, only one news story noted that the ropinirole trials were “relatively short” in duration (the longest was 36 weeks), despite the fact that many people would use the drug for years or even a lifetime.

## Suggestions for How the Media Could Do Better

Unfortunately, there is no obvious way to distinguish information from infomercial. In Table 1, we highlight clues that should alert journalists to the presence of disease mongering, and suggest some things they can do to expose these efforts.

First, journalists should be very wary when confronted with a new or expanded disease affecting large numbers of people. If a disease is common and very bothersome, it is hard to believe that no one would have noticed it before. Prevalence estimates are easy to exaggerate by broadening the definition of disease. Journalists need to ask exactly how the disease is being defined, whether the diagnostic criteria were used appropriately, and whether the study sample truly represents the general population (e.g., patients at an insomnia clinic cannot be taken to represent the general public).

Journalists should also reflexively question whether more diagnosis is always a good thing. Simply labeling people with disease has negative consequences [ 21]. Similarly, journalists should question the assumption that treatment always makes sense. Medical treatments always involve trade-offs; people with mild symptoms have little to gain, and treatment may end up causing more harm than good.

Finally, instead of extreme, unrepresentative anecdotes about miracle cures, journalists should help readers understand how well the treatment works (e.g., what is the chance that I will feel better if I take the medicine versus if I do not?) and what problems it might cause (e.g., whether I might be trading less restless legs for daytime nausea, dizziness, and somnolence).

## Conclusion

The news coverage of restless legs syndrome is disturbing. It exaggerated the prevalence of disease and the need for treatment, and failed to consider the problems of overdiagnosis. In essence, the media seemed to have been co-opted into the disease-mongering process. Although our review was limited to the coverage of a single disease promotion campaign, we think it is likely that our findings would apply to others. It is easy to understand why the media would be attracted to disease promotion stories and why they would be covered uncritically. The stories are full of drama: a huge but unrecognized public health crisis, compelling personal anecdotes, uncaring or ignorant doctors, and miracle cures.

The problem lies in presenting just one side of the story. There may be no public health crisis, the compelling stories may not represent the typical experience of people with the condition, the doctors may be wise not to invoke a new diagnosis for vague symptoms that may have a more plausible explanation, the cures are far from miraculous, and healthy people may be getting hurt.

We think the media could report medical news without reinforcing disease promotion efforts by approaching stories like “restless legs” with a greater degree of skepticism. After all, their job is to inform readers, not to make them sick.

**What Is Restless Legs Syndrome?**

The diagnosis of restless legs syndrome requires the presence of the following four criteria [ 4]:

- An urge to move the legs due to an unpleasant feeling in the legs.
- Onset or worsening of symptoms when at rest or not moving around frequently.
- Partial or complete relief by movement (e.g., walking) for as long as the movement continues.
- Symptoms that occur primarily at night and that can interfere with sleep or rest.

The severity of disease is judged by the frequency of these symptoms, which can range from less than once a month to many times a day. Recommended treatments include stretching exercises and less caffeine for intermittent disease and various prescription drugs (e.g., benzodiazepines and dopamine agonists) for daily symptoms [ 5].

## Abbreviation

FDA: Food and Drug Administration
